# Assessing the transmissibility of epidemics involving epidemic zoning

**DOI:** 10.1186/s12879-023-08205-z

**Published:** 2023-04-18

**Authors:** Baoyin Yuan, Rui Liu, Sanyi Tang

**Affiliations:** 1 School of Mathematics, South China University of Technology, Guangzhou, 510640 China; 2 Pazhou Lab, Guangzhou, 510330 China; 3 School of Mathematics and Statistics, Shaanxi Normal University, Xi’an 710119, China

**Keywords:** Statistical model, Renewal equation, Epidemic zoning, Epidemiology

## Abstract

**Background:**

Epidemic zoning is an important option in a series of measures for the prevention and control of infectious diseases. We aim to accurately assess the disease transmission process by considering the epidemic zoning, and we take two epidemics with distinct outbreak sizes as an example, i.e., the Xi’an epidemic in late 2021 and the Shanghai epidemic in early 2022.

**Methods:**

For the two epidemics, the total cases were clearly distinguished by their reporting zone and the Bernoulli counting process was used to describe whether one infected case in society would be reported in control zones or not. Assuming the imperfect or perfect isolation policy in control zones, the transmission processes are respectively simulated by the adjusted renewal equation with case importation, which can be derived on the basis of the Bellman-Harris branching theory. The likelihood function containing unknown parameters is then constructed by assuming the daily number of new cases reported in control zones follows a Poisson distribution. All the unknown parameters were obtained by the maximum likelihood estimation.

**Results:**

For both epidemics, the internal infections characterized by subcritical transmission within the control zones were verified, and the median control reproduction numbers were estimated as 0.403 (95% confidence interval (CI): 0.352, 0.459) in Xi’an epidemic and 0.727 (95% CI: 0.724, 0.730) in Shanghai epidemic, respectively. In addition, although the detection rate of social cases quickly increased to 100% during the decline period of daily new cases until the end of the epidemic, the detection rate in Xi’an was significantly higher than that in Shanghai in the previous period.

**Conclusions:**

The comparative analysis of the two epidemics with different consequences highlights the role of the higher detection rate of social cases since the beginning of the epidemic and the reduced transmission risk in control zones throughout the outbreak. Strengthening the detection of social infection and strictly implementing the isolation policy are of great significance to avoid a larger-scale epidemic.

**Supplementary Information:**

The online version contains supplementary material available at 10.1186/s12879-023-08205-z.

## Background

Multiple human cases of novel coronavirus disease 2019 (COVID-19) caused by severe acute respiratory syndrome coronavirus 2 (SARS-CoV-2) were first reported in Wuhan, China, from late 2019 to early 2020, although the natural source of the pathogen remains unknown [1]. Since then, more cases have also been identified in other countries or regions of the world, and the trend of transmission has worsened. The World Health Organization (WHO) declared the COVID-19 outbreak a pandemic on 11 March 2020 [2]. As of November 2022, the global spread of COVID-19 has caused more than 631 million human infections and 6.5 million deaths worldwide [3]. At the same time, with the large-scale spread of COVID-19, the continuous mutation of the SARS-CoV-2 genome has evolved into multiple virus variants, labeled the Alpha, Beta, Gamma, Delta and Omicron variants by the WHO in chronological order of discovery [4].

To prevent or delay the spread of the disease, different countries have adopted different control strategies based on their unique sociopolitical backgrounds [5]. In addition to developing new drugs to relieve the symptoms of infected individuals, nonpharmaceutical interventions (NPIs) and vaccination are the most effective protection measures for vulnerable populations in epidemic prevention and control [6]. Given that the enhanced transmissibility and weakening lethality of SARS-CoV-2 variants have led to a continuously increased infection scale and a reduced mortality rate due to infection [7], some countries have adjusted their strategies to live with COVID [5].

However, adhering to a strict and strong strategic policy of “dynamic zero COVID”, COVID-19 has never become uncontrollable nationwide in China. Strict entry quarantine, repeated nucleic acid tests, and even enforcing the lockdown of an entire high-risk city have been commonly used in past local outbreaks. Such strict measures have indeed succeeded in clearing infections and thus resulted in an anti-epidemic situation with Chinese characteristics [8–11]. To reduce the adverse impact on economic life caused by the frequent implementation of the above measures, the government has constantly adjusted the available options as well [12]. In particular, similar to green zoning in Europe [13, 14], the division of the administrative region as a “closed zone”, “control zone” and “prevention zone” has been employed in China [15]. The closed zones are the communities where the infected individuals live and the surrounding areas with frequent activities of the infections. Regional closure, staying at home and door-to-door service are implemented in closed zones. The control zone includes regions such as workplaces and activity areas, where infected individuals visit two days before they develop symptoms or test positive. In control zones, people cannot leave the area, and gatherings are strictly prohibited. The prevention zones are the areas outside the closed zone and control zones within a specific administrative region, where gatherings are strictly limited. Throughout the article, we will collectively refer to the closed zones and the control zones as the control zone. In epidemic practice, once a new case is reported, the control zone would be expanded to cover all the consequent close contacts to ensure that any potential infection is epidemiologically controlled. Following epidemic zoning, new cases can also be reported in a more detailed manner. Therefore, to accurately analyze transmission dynamics, it is necessary to reasonably simulate this dynamic division of epidemic zones when establishing infectious disease models. It is worth noting that China has greatly eased epidemic restrictions on travel and production related to COVID-19 in the late 2022. As a result, the identification and categorization of infected groups are abandoned due to the canceling of the large-scale nucleic acid testing. In spite of the policy adjustment, we believe that the retrospective analysis of epidemic zoning is still of epidemiological significance, thus enriching the modeling methods of infectious diseases.

At present, the epidemic zones in infectious disease models are mostly described in the form of a network. The importance of containment zones within the city in containing outbreaks of either animal infectious diseases or human infectious diseases has been verified by using a dynamical network-based infectious disease model [16, 17]. Liu et al. [18] proposed the method of landscape network entropy, which is a model-free method using only the topological structure of the district network and daily new case data, and they successfully detected the early warning signals of COVID-19 outbreaks. Hunter et al. [19] analyzed the influence of commuters between towns on disease spread by dividing a region into a network of towns. For the epidemiological control of infected cases, the susceptible–infectious–removed (SIR) model and its variants are commonly used in modeling the transfer of infected individuals between different population groups. Tang et al. [20] quantified the effectiveness of quarantine and isolation by means of an adjusted susceptible–exposed–infectious–removed (SEIR) model that took the isolation of cases into account. A novel SEIR-testing, tracing and isolation (TTI) model has been proposed to assess the effectiveness of other NPIs, where the individuals who are diagnosed would be isolated [21].

Although the above models effectively solve the problems they were designed to, the construction of the models requires sufficient epidemiological knowledge and modeling skills. As Bertozzi et al. [22] emphasized, parsimonious models have unique advantages in understanding epidemics, now that they are characterized by simplicity and the capacity to isolate key epidemiological topics of interest. In the current study, we develop a novel parsimonious model that takes epidemic zoning into account, thereby accurately assessing the time-varying transmission risk of the epidemic and providing policy-relevant insights into its course.

## Materials and methods

To identify the epidemiological difference behind the different outbreak sizes, we developed a parsimonious model to accurately assess the disease transmission process by taking into account the epidemic zoning. First, we derived the adjusted renewal equation with continuous case importation by virtue of the Bellman-Harris branching theory, and the derivation procedures are detailed in the appendix. The adjusted renewal equation can be used to describe the iterative process of diseases transmission with case migration between zones. Second, based on the adjusted renewal equation, we built a novel transmission model with epidemic zoning, and we comparatively analyzed the transmission dynamics of two COVID-19 epidemics involving epidemic zoning, so that the driving factors causing the varying outbreak sizes can be discovered.

### Transmission model with epidemic zoning

Let ${I}_{t}^{s}$ be the total number of individuals who are infected in society. Because of the imperfect coverage of case ascertainment efforts, only part of ${I}_{t}^{s}$ would be reported as social cases, i.e., ${I}_{t}^{sr}$. On the other hand, the close tracing of newly confirmed cases led to the expansion of the control zone, which brings the unidentified cases ${I}_{t}^{sc}$ into epidemic control. We assume that the enlarged control zone can cover all potentially infected individuals except the reported cases so that ${I}_{t}^{sr}$ and ${I}_{t}^{sc}$ make up all the socially infected cases ${I}_{t}^{s}$. If we denote a time-varying function ${\beta }_{t}$ as the proportion of the reported cases among all the social infections, then the daily number of unreported cases ${I}_{t}^{sc}$ can be assumed to follow the negative binomial distribution with the reported cases ${I}_{t}^{sr}$, i.e.,

1 $$I_t^{sc} \sim \text{NB}(I_t^{sr},\beta_{t}).$$

Here, the function ${\beta }_{t}$ can be understood as the detection rate of infected cases in society following its definition.

**Fig. 1:**
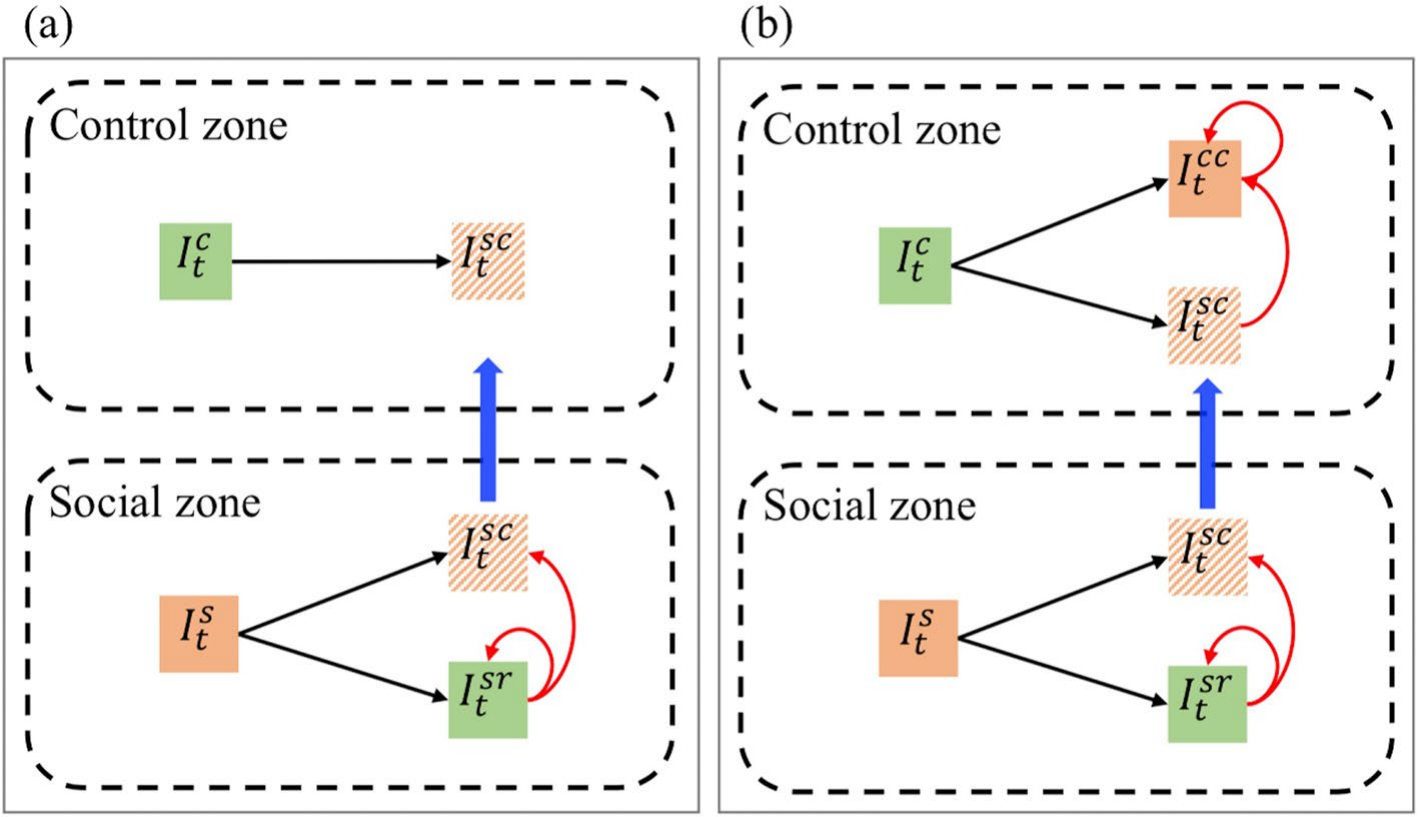
Scheme plot of case transmission between the control zone and the social zone. The left panel (**a**) corresponds to Scenario (a) with a perfect isolation policy; the right panel (**b**) corresponds to Scenario (b) with an imperfect isolation policy. The blue arrow indicates that the cases are transferred from the social zone to the control zone due to epidemiological control. The red arrow represents the transmission direction, i.e., the individual indicated by the arrow is infected by the individual behind the arrow. The green color in the box means that the population number is known; the orange color means that the population number is not directly known. The black arrow refers to the inclusion relation of the case classification

For the control zone, the source of cases can be classified into two scenarios according to whether there is internal infection: (a) perfect isolation policy and (b) imperfect isolation policy, which are shown in Fig. 1.

### Scenario (a): perfect isolation policy

When the implementation of prevention and control measures in the control zone can completely isolate all infected individuals, no internal infections occur. That is, the newly confined cases ${I}_{t}^{sc}$ within society that are brought under control on day $t$ are actually equal to the total number of reported cases that are in control ${I}_{t}^{c}$. In this way, all cases reported in the social and control zones are sourced from social infections. Since the cases ${I}_{t}^{sc}$ did not test positive socially prior to being brought under control, it is reasonable to assume that they are not contagious during their stay in society. Let ${R}_{t}^{{s}_{1}}$ denote the effective reproduction number in society, and let ${g}_{\tau }$ denote the probability density function of the serial interval (the same below). Thus, the transmission process can be expressed as.

2 $$E(I_t^{sr}+I_t^c)=R_t^{s_1}\bullet\int_{\tau=0}^tI_{t-\tau}^{sr}g_\tau d_\tau.$$

Here, the notation of $E$ refers to the expectation and it is used throughout the text.

### Scenario (b): imperfect isolation policy

Internal infections are bound to arise when isolation measures for infected individuals are not perfect, and thus, the reported cases in control ${I}_{t}^{c}$ can be further classified into ${I}_{t}^{sc}$, who are individuals infected in society and reported to be under control, and ${I}_{t}^{cc}$, who are individuals infected in control zones and reported in control zones, i.e., the cases of internal infections within the control zones.

Considering that two types of epidemic prevention and control measures with different intensities are implemented in the control zone and in the social zone, the contact patterns between individuals are also different. In control zones, the strictest measures were taken throughout the epidemic, so the constant transmission intensity of infectious individuals over time can be assumed, denoted as ${R}^{c}$. Thus, the propagation process in control zones can be characterized by the adjusted renewal equation as follows:

3 $$E(I_t^{sr}+I_t^{sc})=R^c\bullet\int_{\tau=0}^tI_{t-\tau}^cg_\tau d_\tau$$

In the social zone, individuals who have not been diagnosed as positive can still move freely, but with the development of the epidemic, the scope of movement will change, so a time-varying transmission intensity can be assumed, denoted as ${R}_{t}^{{s}_{2}}$ (here, the superscript ${s}_{2}$ is to distinguish it from the social reproduction number ${R}_{t}^{{s}_{1}}$ in Scenario (a)). If we maintain the assumption that ${I}_{t}^{sc}$ cannot infect other susceptible individuals before being controlled, the propagation process in society can be written as

4 $$E(I_t^{sr}+I_t^{sc})=R{}_t^{s_2}\bullet\int_{\tau=0}^tI_{t-\tau}^{sr}g_\tau d_\tau$$

### Parameter estimation

Next, we solve for all the unknown parameters in the above models. In Scenario (a), both ${I}_{t}^{sr}$ and ${I}_{t}^{c}$ are known data, so ${R}_{t}^{{s}_{1}}$ as a function of time can be calculated by setting the parameter values of the distribution ${g}_{\tau }$.

However, for Scenario (b), we employ the maximum likelihood technique to estimate the unknown parameters. Assuming that the daily number of new cases reported in control zones follows a Poisson distribution with $E\left({I}_{t}^{c}\right)$ as the expectation, the following likelihood can be constructed:

5 $$L(\Theta\left|I_t^C,I_t^{sr},\right.\sigma)=\prod\nolimits_{t=1}^T\;\frac{\text{exp}\left(-E\left(I_t^c\right)\right)\cdot E{\left(I_t^c\right)}^{I_t^C}}{I_t^c!},$$

where $\sigma$ is the preset distribution parameter of ${g}_{\tau }$ and $\Theta$ contains all unknown parameters that need to be estimated. Here, for the time-varying function ${\beta }_{t}$, we do not directly estimate its value at each time point but resort to the B-spline with fewer unknown parameters to approximate the ${\beta }_{t}$ curve [23, 24].

With the corrected Akaike information criterion (AICc) as the criterion for model selection, the technique of maximum likelihood estimation (MLE) was employed to obtain the optimal estimates of ${\beta }_{t}$ and ${R}^{c}$ on the platform of R software [25]. In sequence, the unobserved cases ${I}_{t}^{sc}$ can be generated following Eq. (1), and immediately, the effective reproduction number in society ${R}_{t}^{{s}_{2}}$ can be calculated for each day. All the codes to perform the estimation and calculation are available from: https://github.com/baoyinyuan/EpidemicZoning.git.

### Two COVID-19 epidemics

In this study, we applied our new transmission models with epidemic zoning to two epidemics with different outbreak sizes, i.e., the Xi’an epidemic from December 09, 2021, to January 17, 2022, and the Shanghai outbreak from March 16, 2022, to June 01, 2022. In both epidemics, epidemic zoning according to transmission risk was implemented, i.e., the cases were reported by their ascertainment areas. Throughout this study, we categorized the cases that were detected in the closed zone or control zone as the cases that were in control zones, and the cases that were detected in the prevention zone or other areas as cases in society. Following this categorization rule, we collected the daily number of reported cases in control zones and in society for both epidemics from the local government website, which are available from: https://github.com/baoyinyuan/EpidemicZoning.git. In addition, since the Xi’an epidemic was caused by the SARS-CoV-2 Delta variant and the Shanghai epidemic was caused by the SARS-CoV-2 Omicron variant, the distribution parameters of serial intervals were set separately. Specifically, a mean of 3.00 days (standard deviation (SD) 2.48 days) for the serial interval distribution of Delta and a mean of 2.75 days (SD 2.53 days) for that of Omicron were employed [7, 26].

## Results

**Fig. 2:**
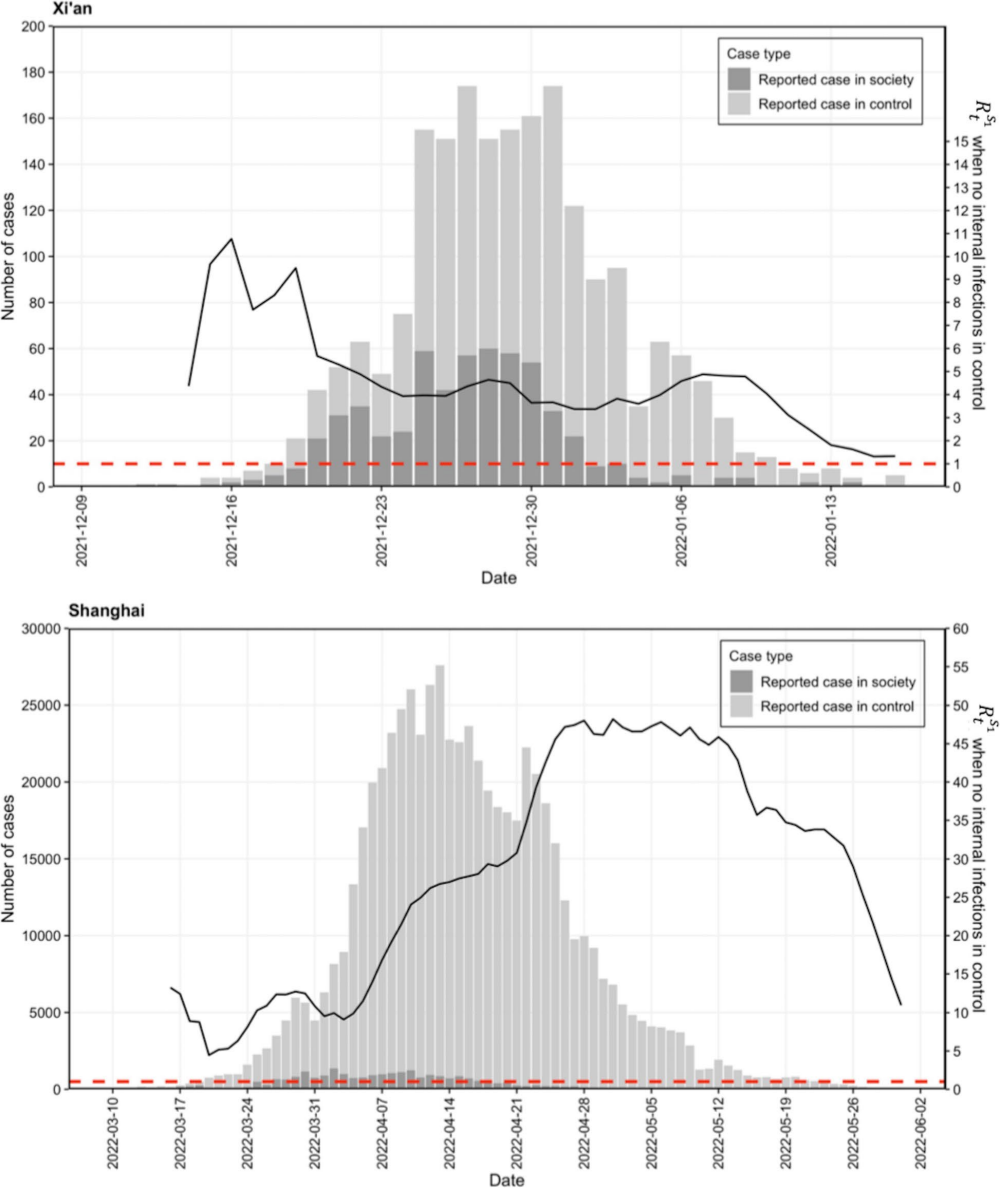
The panel at the top corresponds to the epidemic in Xi’an, and the panel at the bottom corresponds to the epidemic in Shanghai. Bars in light gray and in dark gray represent the number of daily reported cases in control zones and in society, respectively. The black curve is the effective reproduction number ${R}_{t}^{{s}_{1}}$ through time $t$ in society, which is calculated by assuming that there are no internal infections in the control zone, i.e., the scenario (a) in Fig. 1. The size of ${R}_{t}^{{s}_{1}}$ is measured by the right y-axis. When ${R}_{t}^{{s}_{1}}$ is above the threshold value of one, i.e., the horizontal dashed line in red, the epidemic shows an upward trend; otherwise, it is under control

We applied the model framework proposed above to two COVID-19 outbreaks in China, i.e., the Xi’an epidemic and the Shanghai epidemic. In the Xi’an epidemic, a total of 2054 cases were reported, and the Shanghai epidemic caused the largest number of infections since the Wuhan outbreak in early 2020, with a total of more than 627,000 reported cases. Although the outbreak intensity of the two epidemics, including the magnitude of the number of infected individuals and the duration of the outbreak, were very different, the reporting locations of the citywide cases were divided into control zones and social zones during both epidemics. Thus, for the two epidemics, the epidemic curves of the reported cases per day in control zones and in society are shown in the bar plot in Fig. 2. In the Xi’an epidemic, nearly 30% of all cases were reported in society, and in the declining stage after the epidemic peak, there were fewer cases reported in society compared to the previous rising stage. In the Shanghai epidemic, only 4% of cases were reported in society, and similarly, most social cases were distributed in the early stage of epidemic growth.

In Scenario (a), which assumed a perfect isolation policy in control zones, there were no internal infections in the control zones for the two epidemics. Under this assumption, although there were cases reported in the control zones, these cases must have been infected in society before they were epidemiologically controlled. The effective reproduction number ${R}_{t}^{{s}_{1}}$ describes the time-varying transmissibility in society over time, as shown in Fig. 2. The values of ${R}_{t}^{{s}_{1}}$ for both epidemics were directly calculated following Eq. (1). In the Xi’an epidemic, the resulting ${R}_{t}^{{s}_{1}}$ fluctuated between 6 and 10 in the early stage before December 20, 2021, and then until the last day of the epidemic, ${R}_{t}^{{s}_{1}}$ showed a slow downward trend but failed to decrease below the threshold of one. A different curve shape of ${R}_{t}^{{s}_{1}}$ was observed in the Shanghai epidemic. After a fluctuation of approximately ~ 10 in the early stage, the value of ${R}_{t}^{{s}_{1}}$ rapidly increased to the highest level at approximately 50 on April 26, 2022, and continued to drop to ~ 10 after two weeks.

**Fig. 3:**
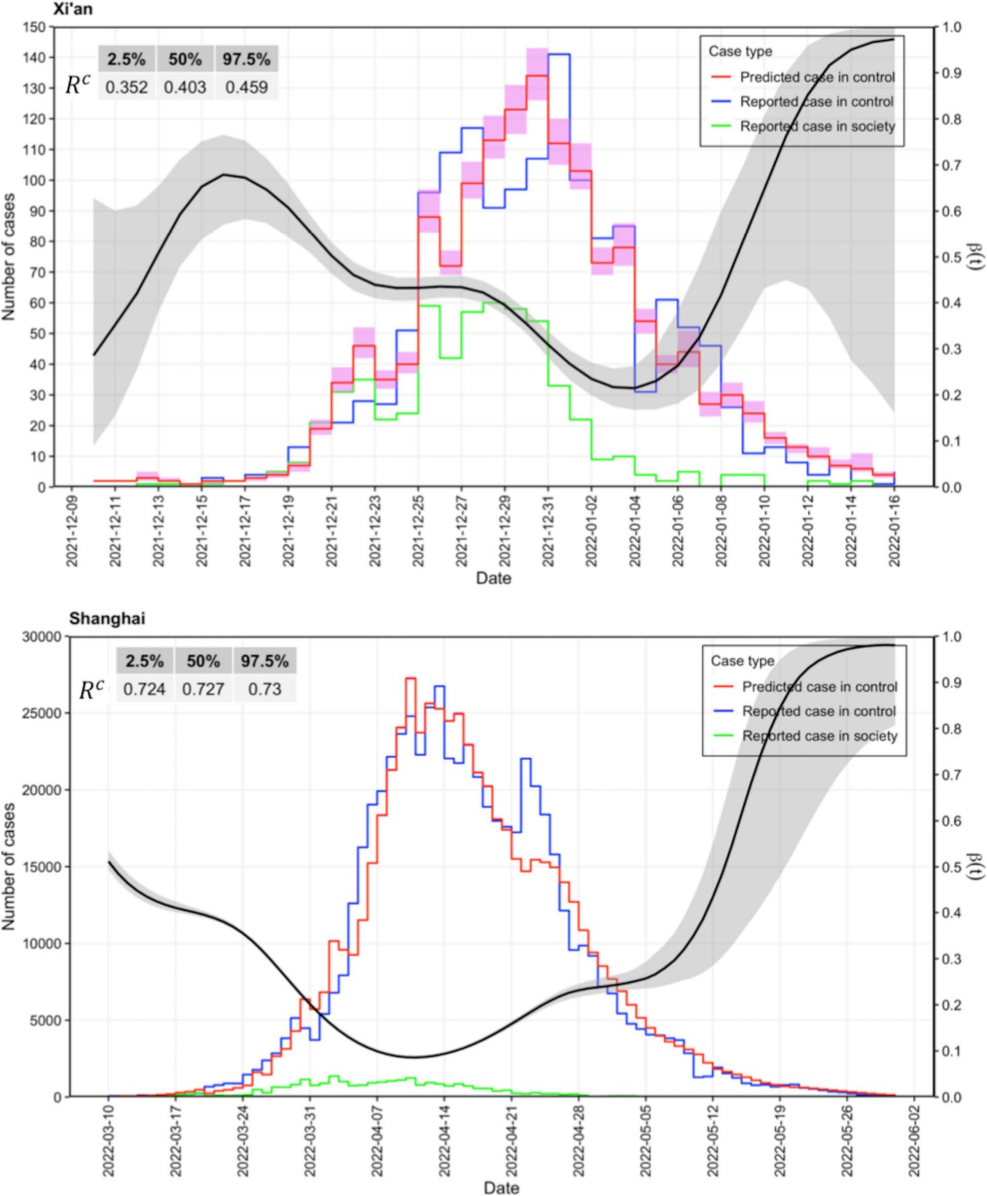
The panel at the top corresponds to the epidemic in Xi’an, and the panel at the bottom corresponds to the epidemic in Shanghai. The step lines represent the change in the daily case counts over time, in which blue is the reported case counts in the control zone, green is the reported case counts in society, and the red line and light red area are the median and 95% confidence interval of the case counts in the control zone predicted by the model. The table in the upper-left corner corresponds to the estimated reproduction number ${R}^{c}$ in the control zone. The black curve represents the time-varying proportion $\beta (t)$ of daily reported cases in society among all the daily infections in society. The size of $\beta (t)$ is measured by the right y-axis

**Fig. 4:**
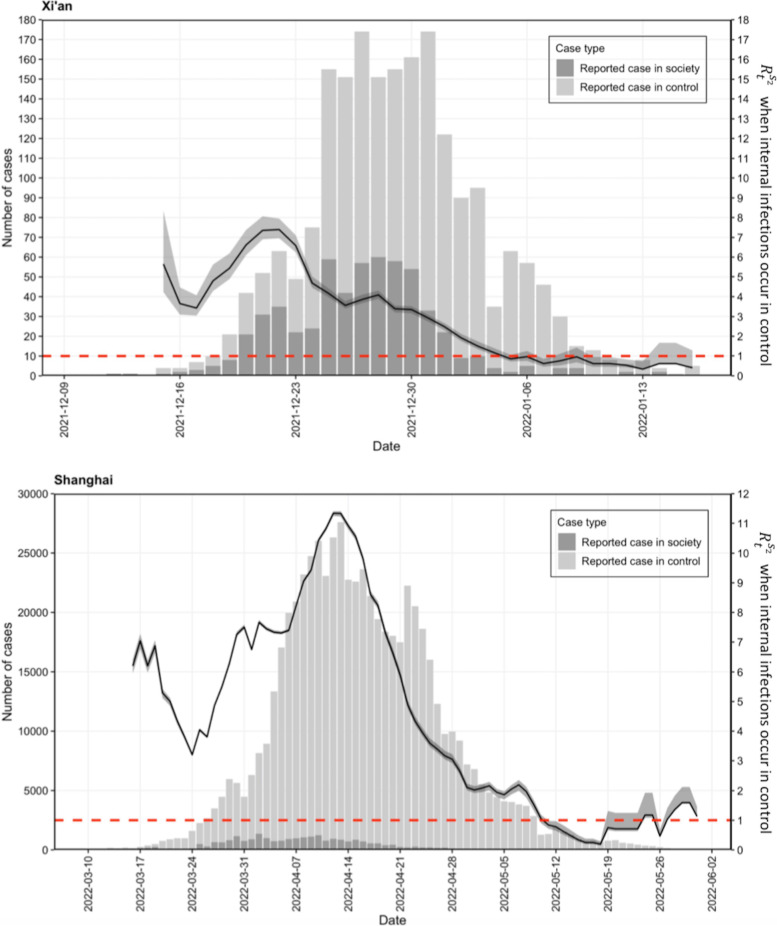
The top panel corresponds to the epidemic in Xi’an, and the bottom panel corresponds to the epidemic in Shanghai. Bars in light gray and in dark gray represent the number of daily reported cases in control zones and in society, respectively. The black curve is the median effective reproduction number ${R}_{t}^{{s}_{2}}$ through time $t$ in society, which is calculated by assuming that there are internal infections in the control zone. The gray is the 95% confidence interval for the predicted ${R}_{t}^{{s}_{2}}$. The size of ${R}_{t}^{{s}_{2}}$ is measured by the right y-axis. When ${R}_{t}^{{s}_{2}}$ is above the threshold value of one, i.e., the horizontal dashed line in red, the epidemic shows an upward trend; otherwise, it is under control

Alternatively, internal infections did occur in the control zones for both epidemics due to the imperfect isolation policy. That is, some of the reported cases in the control zones were from social infections, while others were due to internal infections in the control zones. Considering the different population contact patterns in the control zones and the social zone, the differential transmissibility of the two zones was quantified separately for the two outbreaks, as shown in Figs. 3 and 4. The constant control reproduction numbers ${R}^{c}$ in Xi’an and Shanghai were estimated to have medians of 0.403 (95% confidence interval (CI): 0.352, 0.459) and 0.727 (95% CI: 0.724, 0.730), respectively. In contrast, Fig. 4 presents the time-varying reproduction number ${R}_{t}^{{s}_{2}}$ in society for both epidemics. In the Xi’an epidemic, in addition to the large value of ${R}_{t}^{{s}_{2}}$ in the first week, the overall upward trend remained, and the median ${R}_{t}^{{s}_{2}}$ fell below the threshold value on January 9, 2022. This is highly consistent with the decline in the reported case number in society, with no consecutive socially reported cases since January 9^th^. For the Shanghai epidemic, in the first 10 days, ${R}_{t}^{{s}_{2}}$ fluctuated from 7 to approximately 3.2 and then rapidly increased to a maximum of 11 on April 12, 2022. This was also the day when the daily number of total reported cases and the daily number of reported social cases reached their peak. Since then, ${R}_{t}^{{s}_{2}}$ decreased to below the threshold value of one on May 10, 2022. During the decline period of ${R}_{t}^{{s}_{2}}$, the number of reported social cases also decreased from a few hundred cases per day to sporadic single-digit cases.

In Fig. 3, the daily values of $\beta (t)$ as the numerical proportion of reported social cases among all social infections were estimated to be an intermediate variable for calculating the reproduction number in society. Estimates of $\beta (t)$ for both epidemics exhibited greater uncertainty in the early and late stages of the outbreak due to the reduced number of reported social cases. For the intermediate stage of the Xi’an epidemic from December 15, 2021, to January 08, 2022, a more accurate estimate of $\beta (t)$ was obtained with a narrower CI (confidence interval). From December 10, 2021, $\beta (t)$ grew to the maximum of 0.7 within six days and then gradually decreased to the minimum of 0.21 on January 4, 2022; this phase was also the period when ${R}_{t}^{{s}_{2}}$ gradually declined. Subsequently, the proportion rapidly increased to a level near one; that is, at the end of the epidemic, all social infections were discovered and reported in society. However, during the Shanghai epidemic, $\beta (t)$ showed a very different curve shape. The proportion dropped continuously from 0.5 at the beginning to approximately 0.1 on April 10, 2022, when the epidemic curves peaked. What followed was a period of slow growth to almost 0.25, and it remained there for approximately one week until May 05. Since then, with an increasing number of days that reported zero new social cases, the estimates of $\beta (t)$ had greater uncertainty, and by the end of the epidemic, $\beta (t)$ increased again to approximately one. That is, in the late stage of the two epidemics, the detection rate of social cases reached the highest level of nearly 100%.

## Discussion

In this study, we proposed a novel model framework to quantify the transmissibility of infectious diseases when the division of reported cases into controlled cases and social cases is essential. The model was applied to two COVID-19 epidemics with different magnitudes of case counts in China, i.e., the Xi’an epidemic and Shanghai epidemic. According to whether there were internal infections in the control zone, we quantified the reproduction number of the epidemic. In the scenario without internal infection in control zones, the Shanghai epidemic showed an extremely large social reproduction number over time, while the reproduction number of the Xi’an epidemic was relatively small, but until the end of the epidemic, the social reproduction numbers corresponding to both epidemics did not decrease below the threshold of one. The model results seem to verify the inevitability of internal infection within control zones.

However, once the existence of internal infections in control zones was considered, both our models presented sound effective reproduction number curves over time in society, which accurately reflected the development trend of each of the two epidemics. The social reproduction number in Xi’an showed a downward trend until it was below the threshold of one since the beginning of the epidemic, while Shanghai’s social reproduction number experienced a continuous growth phase for nearly three weeks and then gradually decreased to less than one. In the control zone, even if internal infections could not be eliminated, the estimated reproduction numbers for both epidemics were less than one. Furthermore, we estimated the time-varying proportion of reported cases in society among all the cases who were infected in society. The curve shapes of the proportions were very different in the Xi’an epidemic and the Shanghai epidemic; on average, a larger proportion of social infections were ascertained in society in Xi’an.

There are three lessons to be learned from our exercise. First, by virtue of the Bellman-Harris branching theory [27–29], we derived an adjusted renewal equation that included continuous case importation, and based on this equation, we constructed a new model framework to cover the case migration between epidemic zones. The model framework provides a very useful analysis tool to accurately quantify the disease transmission when epidemic zoning is involved. Moreover, the model framework is not designed to a specific outbreak, but is featured with a good generalizability to all infectious disease outbreaks involving epidemic zoning. Second, for both the Xi’an epidemic and the Shanghai epidemic, the subcritical transmission process did exist within the control zone. Moreover, the smaller control reproduction number indicates stricter implementation of isolation measures in Xi’an than in Shanghai, thus avoiding larger-scale internal infections in the control zones. Third, the curve of $\beta (t)$, which is non-directly measurable, helps us better understand the control efforts of the two cities. The difference in curve shape of $\beta (t)$ between the two epidemics also highlights the importance of a more effective case detection effort in the early stage before the epidemic develops into the exponential growth. When the epidemic scale is small, the case detection efforts make it easier to pinpoint infected cases, thereby increasing the detection rate of cases in society, while during the rapid growth period of the epidemic, the government is more inclined to enlarge the scope of the control zone to bring more potential infections under control within the shortest time. Although the expansion of the control zones can reduce the infection sources in society more quickly, it will also affect more uninfected individuals. Therefore, more emphasis should be placed on the efficientidentification of social cases in the early stage of the epidemic.

The present study was not free from limitations. First, we did not consider the impact of case reporting errors on the modeling analysis. Under current nucleic acid detection capability, it takes approximately one day for routine testing to obtain results from sampling. However, frequent mass screening can substantially delay the reporting of sampling results as the outbreak grows larger. Second, we assumed time-invariant distribution parameters of the serial interval, but the shortened mean length of the serial interval during the epidemic has been reported [30]. Thus, the reproduction number in our modeling must have been underestimated, especially in the late period of the epidemic. Third, the case data we used only included locally reported cases, involving no imported cases from abroad.

In summary, we retrospectively analyzed the transmission dynamics of the Xi’an epidemic in late 2021 and the Shanghai epidemic in April 2022 by using our newly derived renewal equation with continuous case importation. The transmissibility in the epidemiologically control zones and the uncontrolled areas, i.e., the social zones, has been accurately quantified. In both epidemics, internal infections characterized by a subcritical transmission process within the control zones were confirmed by the model. To prevent a large-scale outbreak of the epidemic, we emphasize that early detection of infected individuals in society is key.

## Supplementary Information

**Additional file 1.**

## Data Availability

The datasets supporting the conclusions of this article have been uploaded as part of the supplementary material. All the codes to perform the estimation and calculation are available from: https://github.com/baoyinyuan/EpidemicZoning.git.
