# Supplementary material for: Assessing the transmissibility of epidemics involving epidemic zoning
Source: BMC Infect Dis. 2023 Apr 18;23:242. doi: 10.1186/s12879-023-08205-z (PMC10111305; doi:10.1186/s12879-023-08205-z)
Supplement: Supplementary file 1 — Additional file 1. [file 12879_2023_8205_MOESM1_ESM.docx]

**Appendix. Adjusted renewal equation with continuous case importation**

**Bellman-Harris process**

For one population who are susceptible to one type of infectious agent, we assume the index case is introduced into this population at time $t=0$. The time that the index case spends to cause other infections can be measured by the random variable $\tau$ with the cumulative distribution function $G(\tau)(=\int_{0}^{\tau} g(t)dt)$. At the moment when one primary case causes a random number of secondary cases according to a probability distribution $q_{n}\equiv P(Y=n)$ with probability generating function $h\left( s \right)\equiv E\left( s^{Y} \right)=\sum_{n=0}^{\infty} q_{n}s^{n}$, the primary case disappears. Each of the first-generation cases behaves, independently of each other and their primary cases, as the primary cases did, i.e., it spends a random time $\tau$ to cause their next-generation cases, produces a random number of secondary cases according to $h\left( s \right)$, and disappears simultaneously. If we denote $X(t)$ the number of infected cases at time $t$, then the stochastic process $\{X\left( t \right), t\geq0\}$is called Bell-Harris branching process [1]

**Bellman-Harris integral equation**

Assume that the time interval between two successive generations of cases is equal to $\tau$. For time $t<\tau$, the process consists of the single index case; for $t\geq\tau$, the number of cases in the process is the sum of the numbers of cases in all subprocess started by the first-generation infections, i.e.,

$X\left( t \right)=\left\{ \begin{matrix} \begin{matrix} 1, & t<\tau\end{matrix} \\ \begin{matrix} \sum_{n=1}^{Y} X^{\left( n \right)}(t-\tau), & t\geq\tau\end{matrix} \end{matrix} \right.$ (1)

where $Y$ is the random variable to describe the offspring distribution, i.e., the number of secondary infections caused by each infected individual. $X^{\left( n \right)}(t-\tau)$ indicates the branching subprocess started by the first-generation cases of index cases at time $\tau$ is independent identically distributed. Let $p_{n}\left( t \right)=P(X(t)=n)$ denote the probability distribution of having $n$ infected cases at time $t$, then $X(t)$ has probability generating function $F\left( s, t \right)=E\left( s^{X} \right)=\sum_{n=0}^{\infty} p_{n}(t)s^{n}$. When $t<\tau$, equivalently, $X\left( t \right)=1$, i.e., the index case has not infected anyone by time $t$, we have $F\left( s, t \right)=p_{0}+p_{1}\cdot s=(1-G(t))\cdot s$; when $t\geq\tau$, the index case has infected some number of secondary cases according to $q_{n}(n>1)$. Thus, removing conditioning on $\tau$, we have the so-called Bellman-Harris integral equation:

$F\left( s, t \right)=\left( 1-G\left( t \right) \right)\cdot s+\int_{\tau=0}^{t} h(F(s, t-\tau))dG(\tau)$. (2)

The second integral term is the direct result of Property 1. [1,2].

**Renewal equation**

- **Property 1**. Suppose that $Y$ is a random variable taking natural-number values with probability generating function $G_{Y}$ and $\{X^{\left( i \right)}, i\geq1\}$ independent of $Y$ is a sequence of random variables taking natural-number values. If $X^{1}, X^{2},\cdots,X^{Y}$are independent and identically distributed with common probability generating function $G_{X}$, then the probability generating function $G_{S_{Y}}$ of $S_{Y}=\sum_{i=1}^{Y} X^{(i)}$ is $G_{S_{Y}}(s)=G_{Y}(G_{X}(s))$, where $s\in U\equiv[0,1]$ is a symbolic argument.

The first moment of $X\left( t \right)$, i.e., the expected number of infected cases at time $t$ can be computed from the pgf $F\left( s, t \right)$ by taking its derivative and evaluating at $s=1$,

$f\left( t \right)\equiv E\left( X\left( t \right) \right)={\frac{\partial F\left( s,t \right)}{\partial s}|}_{s=1}=\sum_{n=1}^{\infty} n\cdot p_{n}(t)$.

On the other hand, we realize that

$F\left( 1,t-\tau\right)=\sum_{n=0}^{\infty} p_{n}\left( t-\tau\right)=1$, and

$\frac{\partial h(s)}{\partial s}=\sum_{n=1}^{\infty} n\cdot q_{n}\cdot s^{n-1}$.

Instantly, we have

$\begin{matrix} {\frac{\partial h\left( F\left( s,t-\tau\right) \right)}{\partial s}|}_{s=1}= & {\sum_{n=1}^{\infty} n\cdot q_{n}\cdot{F\left( s,t-\tau\right)}^{n-1}|}_{s=1} \\ = & \sum_{n=1}^{\infty} n\cdot q_{n}\cdot{F\left( 1,t-\tau\right)}^{n-1} \\ = & \sum_{n=1}^{\infty} n\cdot q_{n} \end{matrix}$,

which is actually the basic reproduction number $R_{0}$.

Thus, according to the B-H integral equation, $f\left( t \right)$ can also be written as follows,

$\begin{matrix} f\left( t \right)={\frac{\partial F\left( s,t \right)}{\partial s}|}_{s=1}= & 1-G\left( t \right)+\int_{\tau=0}^{t} \frac{\partial F(1,t-\tau)}{\partial s}\cdot\frac{\partial h\left( F\left( 1, t-\tau\right) \right)}{\partial s}\cdot g(\tau)d\tau\\ = & 1-G\left( t \right)+R_{0}\cdot\int_{\tau=0}^{t} \frac{\partial F\left( 1,t-\tau\right)}{\partial s}\cdot g\left( \tau\right)d\tau\\ = & 1-G\left( t \right)+R_{0}\cdot\int_{\tau=0}^{t} f\left( t-\tau\right)\cdot g\left( \tau\right)d\tau\end{matrix}$.

The above iterative equation is the renewal equation. If we assume the offspring distribution is time-varying, i.e*.*, $h\left( s, t \right)$, the effective reproduction number $R_{t}$ will appear in place of $R_{0}$, and the more general renewal equation can be obtained following the same deduction process [3],

$f\left( t \right)=1-G\left( t \right)+R_{t}\cdot\int_{\tau=0}^{t} f(t-\tau)\cdot g(\tau)d\tau$. (3.1)

**Adjusted renewal equation to cover imported cases**

Until now, we have assumed that the entire transmission process is triggered by one index case and there are no exogenous cases entering the population under consideration. But for a target population that has personnel communication with other populations outside, the importation of infected individuals is inevitable. In the following, we assume the imported cases possess the same transmission characteristics as the endogenous cases once they enter the target population.

In terms of case importation, we consider several scenarios:

**Scenario 1**: Assume that there are $m$ index cases at time $t=0$, the expected number of infected cases in the future time $t$ would be

$f\left( t \right)=m\cdot\left( 1-G\left( t \right) \right)+R_{t}\cdot\int_{\tau=0}^{t} f(t-\tau)\cdot g(\tau)d\tau$. (4)

**Scenario 2**: Assume that the single index case is introduced into the population at time $t=T$($T>0$), then the starting time of the epidemic would shift time $T$ to the right, i.e.,

$f\left( t \right)=1-G(t-T)+R_{t}\cdot\int_{\tau=0}^{t} f(t-\tau)\cdot g(\tau)d\tau$. (5)

**Scenario 3**: Assume that $\mu(t)$ cases are introduced into the population at different time $t>0$, and these imported cases at the later times possess the same transmission characteristics as the descendant cases produced by the index case at the same time. In this case, the expected number of infected cases in the future time $t$ is

$f\left( t \right)=1-G\left( t \right)+R_{t}\cdot\int_{\tau=0}^{t} f\left( t-\tau\right)\cdot g\left( \tau\right)d\tau+\mu(t)$. (6)

**Left-truncated renewal equation**

For the general renewal equation in continuous form, it can be further decomposed:

$\begin{matrix} f\left( t \right)= & 1-G\left( t \right)+R_{t}\cdot\int_{\tau=t-t_{1}}^{t} f\left( t-\tau\right)\cdot g\left( \tau\right)d\tau+R_{t}\cdot\int_{\tau=0}^{t-t_{1}} f\left( t-\tau\right)\cdot g\left( \tau\right)d\tau\\ = & \Gamma\left( t \right)+R_{t}\cdot\int_{\tau=0}^{t-t_{1}} f\left( t-\tau\right)\cdot g\left( \tau\right)d\tau\end{matrix}$ (7.1)


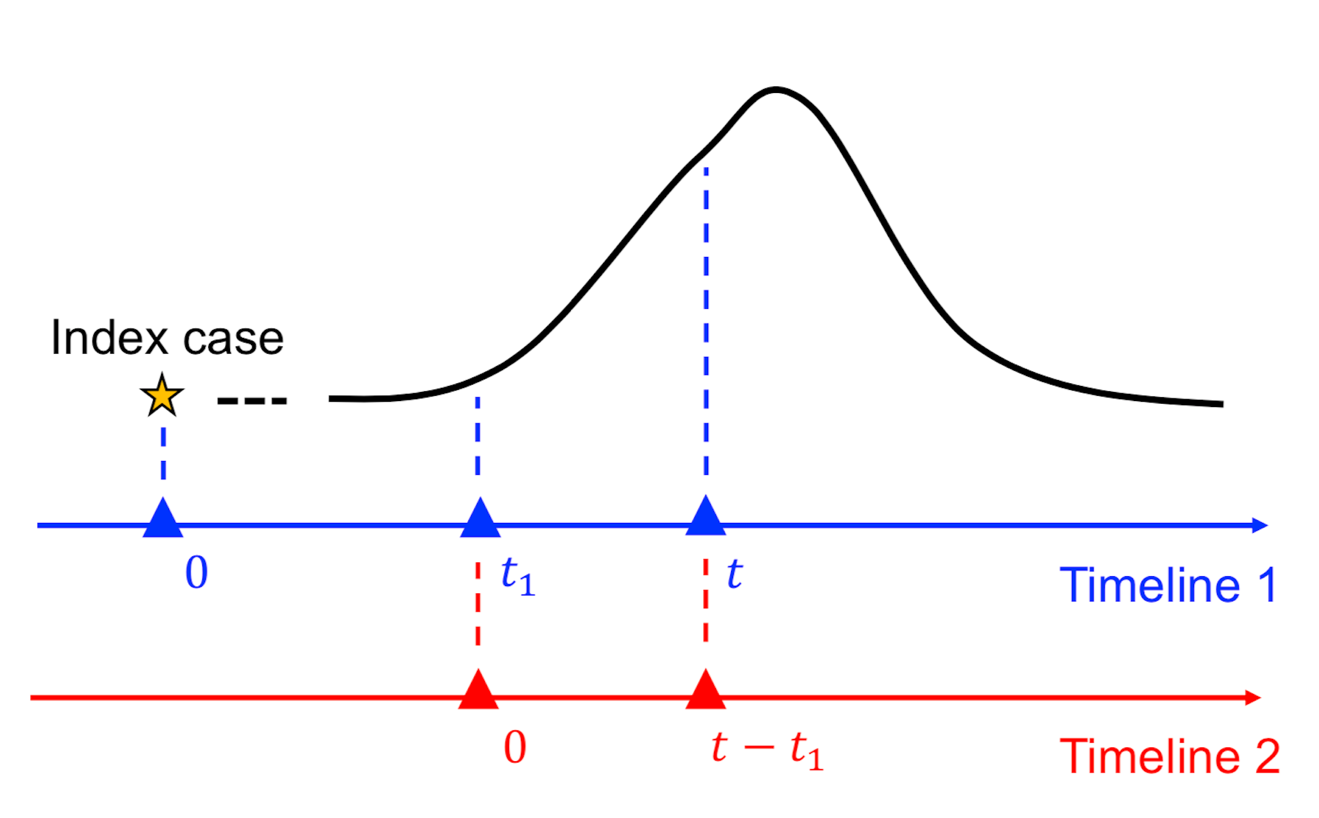


Figure A1. The schematic plot of epidemic curve with two timelines. For the timeline 1 in blue, the time when index case is introduced is the time zero; for the timeline 2 in red, the time $t_{1}$ in timeline 1 is the time zero for epidemic analysis.

If we consider the time when the index case is introduced to be the initial time of epidemic outbreak, i.e., $t=0$ in timeline 1 as shown in Fig. A1., Eq. (3.1) completely describes the propagation process. In addition, let us look into the equivalent renewal equation (7.1), the part to the right side of the last equal sign actually highlights the impact of the two types of cases on the current number of emerging cases at time $t$. $\Gamma\left( t \right)$ describes the impact of cases before $t_{1}$, while the second term with integral describes the impact of cases after $t_{1}$. It is worth noting that the index case is always difficult to be identified in the real epidemic such as COVID outbreak. Epidemiological analysis that does not involve traceability of transmission usually begin at a later time, e.g., $t=t_{1}$, when there is less uncertainty in case reporting. That is, if the cases before $t_{1}$ do not affect the subsequent analysis of the epidemiological problem of interest, the renewal equation can be further simplified as follows,

$f\left( t \right)\cong R_{t}\cdot\int_{\tau=0}^{t-t_{1}} f\left( t-\tau\right)\cdot g\left( \tau\right)d\tau.$ (8)

Now let us shift the timeline 1 to timeline 2 by setting the time $t_{1}$ in timeline 1 as time zero in timeline 2, then time $t$ in timeline 1 becomes time $t-t_{1}$ in timeline 2, but the relative time interval $|t-t_{1}|$ remain the same. As a result, we have the new equation after time shifting

$f\left( t \right)\cong R_{t}\cdot\int_{\tau=0}^{t} f\left( t-\tau\right)\cdot g\left( \tau\right)d\tau.$ (9)

The above equation is actually the commonly used renewal equation in epidemiology for modelling incidence [4-6].

**Final renewal equation with left truncation and case importation**

In all the above analysis, we only consider the continuous evolvement of the number of infected individuals over time. However, case reporting is always discontinuous and binned into hourly counts, daily counts, etc. For the discrete data of case number, the above equation (3.1) can be discretized as:

$f\left( t \right)=1-G\left( t \right)+R_{t}\cdot\sum_{\tau=0}^{t} f(t-\tau)\cdot g(\tau)$, (3.2)

where $t\in\{Z_{+}:1, 2, 3, \cdots\}$. Accordingly, it can also be decomposed as:

$f\left( t \right)=1-G\left( t \right)+R_{t}\cdot\sum_{\tau=t-t_{1}+1}^{t} f\left( t-\tau\right)\cdot g\left( \tau\right)+R_{t}\cdot\sum_{\tau=0}^{t-t_{1}} f\left( t-\tau\right)\cdot g\left( \tau\right)$.

(7.2)

Moreover, the recursive equation can be extended to include importation of exogenous cases $\mu_{t}$, which are assumed to possess the same transmission characteristics as the descendant cases produced by the antecedent cases prior to the current time $t$.

$f\left( t \right)=1-G\left( t \right)+R_{t}\cdot\sum_{\tau=t-t_{1}+1}^{t} f\left( t-\tau\right)\cdot g\left( \tau\right)+R_{t}\cdot\sum_{\tau=0}^{t-t_{1}} f\left( t-\tau\right)\cdot g\left( \tau\right)+\mu_{t}$. (10)

According to our discussion above about the left-truncation of the starting time for epidemic analysis, if the impact of endogenous and exogenous cases before time $t_{1}$ on the current number of cases at time $t$ is negligible, the above equation can also be left truncated at time $t_{1}$ by removing all the terms related to the previous cases prior to $t_{1}$. As a result, we have the simplified renewal equation

$f\left( t \right)\cong R_{t}\cdot\sum_{\tau=0}^{t-t_{1}} f\left( t-\tau\right)\cdot g\left( \tau\right)+\mu_{t}$. (11)

Next, we shift Timeline from 1 to 2 by setting time $t_{1}$ to be the new zero time,

$f\left( t \right)\cong R_{t}\cdot\sum_{\tau=0}^{t} f\left( t-\tau\right)\cdot g\left( \tau\right)+\mu_{t}$. (12)

This is the discrete form of the commonly used renewal equation with case importation in epidemiology.

**Reference**

1. Kimmel M, Axelrod DE. 2002 Branching Processes in Biology. New York: Springer. (doi:10.1007/b97371)
2. Mishra S, Berah T, Mellan TA, Unwin HJT, Vollmer MA, Parag KV, Gandy A, Flaxman S, Bhatt S. 2006 On the derivation of the renewal equation from an age-dependent branching process: an epidemic modelling. *arXiv*. **2006**, 16487v1. (https://doi.org/10.48550/arXiv.2006.16487)
3. Berah T, Mellan TA, Miscouridou X, Mishra S, Parag KV, Pakkanen MS, Bhatt S. 2021 Unifying the effective reproduction number, incidence, and prevalence under a stochastic age-dependent branching process. *arXiv*. **2107**, 05579. (https://arxiv.org/pdf/2107.05579v1)
4. Fraser C, Riley S, Anderson RM, Ferguson NM. 2004 Factors that make an infectious disease outbreak controllable. *Proc. Natl Acad. Sci. USA*. **101**, 6146–6151. (https://doi.org/10.1073/pnas.0307506101)
5. Aldis GK, Roberts MG. 2005 An integral equation model for the control of a smallpox outbreak. *Math. Biosci.* **195**, 1–22. (doi:10.1016/j.mbs.2005.01.006)
6. Green WD, Ferguson NM, Cori A. 2022 Inferring the reproduction number using the renewal equation in heterogeneous epidemics. *J. R. Soc. Interface*. **19**, 20210429. (https://doi.org/10.1098/rsif.2021.0429)
